# Physiological Responses and Post-Exposure Recovery of the Hepatopancreas in Nile Tilapia Following Copper Exposure

**DOI:** 10.3390/toxics14050412

**Published:** 2026-05-09

**Authors:** Xueyi Wu, Wenqi Xie, Zhengfan Chen, Ziyi Jiang, Jiazhe Jiang, Lei Xie, Yongpu Zhang

**Affiliations:** 1 College of Life and Environmental Science, Wenzhou University, Wenzhou 325035, China; 2 Zhejiang Provincial Key Laboratory for Water Environment and Marine Biological Resources Protection, Wenzhou University, Wenzhou 325035, China

**Keywords:** Nile tilapia, copper ions, oxidative stress, gene expression, histology

## Abstract

Copper is a common pollutant in aquatic environments. Excess copper in water can enter aquatic organisms through respiration, feeding, and adsorption, thereby exerting serious adverse effects on their health. In this study, NEW Genetically Improved Farmed (GIFT) Nile tilapia (*Oreochromis niloticus* L.) was used to explore the effect of copper on the hepatopancreas and post-exposure recovery. Acute exposure was simulated via an intraperitoneal injection of 3.75 mg Cu^2+^/kg body mass, while physiological saline injections served as the control. Samples were collected on days 1, 7, 14, and 21 post-exposure to evaluate growth performance, histopathological changes, antioxidant enzyme activities, and the expression of oxidative stress-related genes in the hepatopancreas. The results show that body length and mass increased within 21 days of the injection and copper exposure did not significantly affect fish growth. On day 1 after copper injection, numerous vacuoles appeared in hepatopancreatic tissues. On day 14, congestion and obvious hepatic sinusoids were observed. However, on day 21, the tissue structure showed gradually recovery. Compared to the control group, superoxide dismutase (SOD) activity was significantly higher in the exposed group on days 1, 14, and 21, and *SOD* gene expression was significantly elevated on day 21. Catalase (CAT) activity was significantly higher on day 7, and the expression of the *CAT* gene increased significantly on days 1 and 21. Glutathione peroxidase (GSH-Px) activity decreased significantly on day 7, whereas *GPX* gene expression increased significantly at the same time point. No significant difference in acetylcholinesterase (AChE) activity was observed during the experiment. In conclusion, copper administered via intraperitoneal injections induced significant activation of the antioxidant defense system and histopathological damage in the hepatopancreas of tilapia. Although tissue damage gradually recovered over time, the activation of the antioxidant defense system partially persisted. Ultimately, copper exposure did not significantly affect growth indicators such as body length and mass. These results advance our understanding of copper toxicity in farmed fish and provide a scientific reference for safe aquaculture production.

## 1. Introduction

As the world’s most populous country and a major developing economy, China has experienced unprecedented rapid industrialization and large-scale urbanization in recent decades [1]. Consequently, heavy metal pollution in aquatic environments has become increasingly severe, and copper has emerged as one of the most enriched heavy metals in lakes. Between 1972 and 2017, the total copper concentration in 113 rivers and lakes across Asia averaged 345.85 ± 246.43 μg/L. Copper concentrations increased continuously from the 1970s to the 2000s, peaked in the 2000s, and then declined in the 2010s [2]. From 2006 to 2017, the monitored range of copper concentrations in China’s eight major river basins was 0.01 to 8628 μg/L, and chronic ecological risks caused by copper contamination exceeded 50% in several basins, including the Haihe, Yangtze, Songhua, Pearl, and Yellow Rivers [3]. Therefore, copper has become a major heavy-metal pollutant and one of the primary threats to aquatic organisms in aquatic ecosystems [4,5].

As an accessory factor in many enzymatic processes, copper is an essential trace element for organismal growth and development. However, excessive copper can cause many toxic effects in organisms, and these effects vary among species and developmental stages. For example, the 96 h LC_50_ of copper sulfate in *Labeo rohita* is 0.37 mg/L at embryonic and larval stages, 0.75 mg/L at the swim-up fry stage, 1.07 mg/L at advanced fry stage, and 1.34 mg/L at the fingerling stage, whereas in *Cirrhinus mrigala*, it is 0.48 mg/L at the embryonic and larval stages, 0.94 mg/L at the swim-up fry stage, 1.36 mg/L at the advanced fry stage, and 1.52 mg/L at fingerling stage [6]; the 48 h LC_50_ of copper sulfate in *Pimephales promelas* is 0.90 mg/L [7]. Excess copper can induce various adverse effects [8] such as reduced growth and survival rates [9], damage to the gills and liver [9,10], ribosomal damage [11], abnormal collagen metabolism [12], tissue antioxidant stress [9,13], and impaired reproduction [14].

Oxidative stress damage caused by copper is the most concerning toxicological effect. Under normal conditions, the antioxidant enzyme system, comprising enzymes such as superoxide dismutase (SOD), catalase (CAT), and glutathione peroxidase (GSH-Px), can effectively eliminate reactive oxygen species (ROS) generated by various metabolic activities, including singlet oxygen (1O_2_), superoxide anion radical (O^2−^), hydroxyl radicals (-OH), and hydrogen peroxide (H_2_O_2_), thereby protecting biological macromolecules in animals from free radical damage [15]. The production of ROS and the scavenging capacity of the antioxidant system are in a state of dynamic balance, known as “redox homeostasis” [16]. When the concentration of heavy metals exceeds the safe threshold, the organism produces an excess of ROS that surpasses the scavenging capacity of the antioxidant enzyme system, leading to changes in the activities of various antioxidant enzymes and ultimately exerting toxic effects on the organism [17]. Although a considerable number of studies have confirmed that copper exposure induces oxidative stress, those on the recovery process and underlying mechanisms of oxidative damage after cessation of exposure remain relatively limited. Some studies have suggested that the damage induced by heavy metals may be reversible [18,19,20,21]; however, whether these recovery responses are complete requires further investigation. In addition, acetylcholinesterase (AChE) is a key rate-limiting enzyme in biological nerve conduction. It can accurately reflect the effects of exogenous pollutants on the nervous system and overall physiological homeostasis of organisms [22]. As a widely recognized universal biomarker for aquatic ecotoxicity assessment worldwide, it sensitively indicates physiological damage and health status in fish under heavy metal stress [23].

NEW Genetically Improved Farmed (GIFT) Nile tilapia (*Oreochromis niloticus* L.), belonging to Perciformes, Cichlidae, and *Oreochromis*, is an improved strain developed through hybrid breeding. Due to its clear genetic background, stable traits, and strong environmental adaptability, it has become an ideal experimental model for studies on fish nutrition, stress physiology, and genetic breeding. In addition, intraperitoneal injection serves as an appropriate exposure route for exploring the toxicological mechanisms of toxicants [24]. Although not representative of natural exposure scenarios, intraperitoneal injection is widely used as an exposure route in ecotoxicological studies [25]. Therefore, this study simulates short-term copper exposure via an intraperitoneal injection to investigate the effects of acute copper exposure and subsequent recovery in tilapia. This study aims to improve our understanding of copper toxicological mechanisms, provide theoretical support for aquaculture product safety control, and offer a reference for healthy tilapia farming.

## 2. Materials and Methods

### 2.1. Animals and Experimental Design

Tilapia were purchased from an aquaculture farm in Huzhou, Zhejiang Province, China, regardless of sex. The experimental fish used in this study were 30 days post-hatch (DPH), with a mean body length of 3.24 ± 0.48 cm and a mean body weight of 3.18 ± 1.34 g. In the laboratory, the fish were acclimated for one week in an outdoor pond with dimensions of 3 m × 1 m × 0.7 m (length × width × depth) under natural conditions. In addition, a continuous flow-through culture system was adopted, with tap water as the water source, and the flow rate was controlled at approximately 2 L/min. Fish health was evaluated mainly based on their activity behavior and body surface integrity, including the presence or absence of scale loss and fin erosion. The fish were fed commercial tilapia feed daily at 9:30, and behavioral conditions were monitored. Daily air temperature was recorded using an iButton device (Analog Devices, Wilmington, MA, USA). After acclimation, healthy individuals of similar sizes were randomly divided into two groups (120 fish per group). Copper exposure was simulated via an intraperitoneal injection. The injection concentration was determined based on a study of *Oreochromis niloticus* [25] and our earlier unpublished acute toxicity tests (1/4–1/6 of the 96 h LD_50_). A stock solution of Cu^2+^ (0.15 mg/mL) was prepared by dissolving CuSO_4_·5H_2_O (AR, Shanghai Sinpeuo Fine Chemical Co., Ltd., Shanghai, China). Physiological saline injections served as the control. Before injection, each tilapia was anesthetized with MS-222 (Merck KGaA, Darmstadt, Germany) and weighed. The injection volume was adjusted based on body weight to achieve the target exposure dose of 3.75 mg Cu^2+^/kg body weight. After the injection, the fish were allocated to 2 ponds based on their treatment group. The fish were monitored daily. At 1, 7, 14, and 21 days post-injection, 30 fish were randomly collected from each group. Fish were anesthetized with MS-222 before measuring their body mass, and hepatopancreas mass and length, followed by hepatopancreas dissection. The hepatosomatic index (HSI) was calculated as follows: HSI = hepatopancreas mass/body mass. Five individuals were randomly selected from each sampling group for histological examination, and these samples were fixed in 4% paraformaldehyde for histology, while remaining samples were snap-frozen in liquid nitrogen and stored at −80 °C.

### 2.2. Histopathological Analysis

The fixed tilapia hepatopancreatic tissues were processed using paraffin-embedding procedures. After dehydration, clearing, embedding, and sectioning, tissue sections with a thickness of 5–8 μm were obtained. The paraffin sections were stained with hematoxylin and eosin (H&E), and the histological structures were subsequently observed under a light microscope. Details of the procedure are provided in the Supplementary Materials.

### 2.3. Enzyme Activity Assays

The hepatopancreas samples stored at −80 °C (*n* = 6) were thawed and weighed. Physiological saline was added at a mass-to-volume ratio of 1:9, followed by thorough homogenization on ice.

The total protein content in the hepatopancreas samples was quantified using the Coomassie Brilliant Blue colorimetric method with a commercial Total Protein Assay Kit (Cat No. A045-2-2, Nanjing Jiancheng Bioengineering Institute, Nanjing, China). All procedures were carried out in strict accordance with the manufacturer’s instructions: reagents and samples were added sequentially and incubated at 37 °C, and the absorbance was read at 562 nm for protein concentration calculation based on the standard curve. The activities of SOD (Cat No. A001-3-2), CAT (Cat No. A007-1-1), GSH-Px (Cat No. A005-1-2) and AChE (Cat No. H529-1-2) were assayed using the corresponding commercial kits from the Nanjing Jiancheng Bioengineering Institute. All operations were performed by strictly following the kit protocols. After the sequential addition of the reagents and samples into the reaction system, the mixture was incubated at the set temperature for the required time. Changes in absorbance were then detected at the specified wavelength using a microplate reader, and enzyme activity was calculated using the formula provided in the instructions. The specific formulas are provided in the Supplementary Materials.

### 2.4. Gene Expression

Total RNA was extracted from the hepatopancreatic tissues (*n* = 6) stored at −80 °C using a column-based RNA extraction kit (B518701, Sangon Biotech, Shanghai, China). The six individuals used in this assay were not the same as those used in the histological examination, but they were randomly chosen from the same sampling population as mentioned above. RNA purity and concentration were measured using a NanoDrop 2000 (Thermo Fisher Scientific, Waltham, MA, USA), and RNA integrity was verified via 1% agarose gel electrophoresis. The extracted total RNA was then reverse-transcribed into cDNA using a reverse transcription kit, and quantitative real-time PCR (qPCR) was performed using a fluorescent quantitative PCR kit with Roche Light Cycler 480 (Roche Applied Science, Penzberg, Germany). Details of the commercial kits and corresponding reaction conditions are provided in the Supplementary Materials. *β-actin* was used as the reference gene to determine the relative expression levels of *SOD*, *GPX*, and *CAT* in the hepatopancreas. The primers were obtained from Saddick et al., 2017 [26]. Each sample was analyzed in triplicate, and the mean value was used for analysis. Relative gene expression levels were calculated using the 2^−ΔΔCT^ method.

### 2.5. Statistical Analysis

Statistical analyses of all experimental data were performed using one-way analysis of variance (one-way ANOVA), and multiple comparisons were conducted using the Games-Howell test. Descriptive statistics are presented as mean ± standard error (SE), and differences were considered statistically significant at *p* ≤ 0.05. Data analyses were carried out using SPSS 27 software, and figures were generated using GraphPad Prism 10.

## 3. Results

### 3.1. Environmental Temperature and Morphological Effects

The average air temperature during the experiment was 29.7 °C (Supplementary Figure S1). A temporary decrease to 26.4 °C occurred on day 3 due to a typhoon but it subsequently recovered. Body length, body mass, and hepatopancreas mass increased gradually after Cu^2+^ exposure, with no significant differences between groups (Table 1). No mortality was observed throughout the experiment.

### 3.2. Histological Effects on the Hepatopancreas

**Figure 1:**
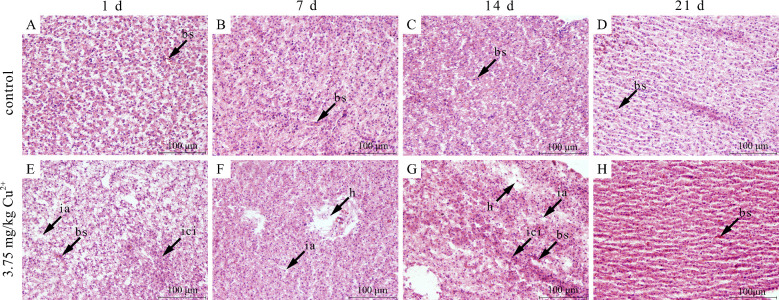
Histological sections of the hepatopancreas in the control group after 1 day (**A**), 7 days (**B**), 14 days (**C**), and 21 days (**D**). Histological sections of the hepatopancreas in the treatment group after exposure to 0.15 mg/kg Cu^2+^ for 24 h (**E**), 7 days (**F**), 14 days (**G**), and 21 days (**H**). bs, blood sinus; ici, inflammatory cell infiltration; h, tissue vacuolation; ia, intercellular space.

Histological observations showed that the hepatopancreatic cells of the control tilapia were evenly spaced and regularly arranged, with relatively large, nearly oval-shaped cell bodies. The nuclei were spherical and centrally located (Figure 1A–D). On day 1 after exposure, an increased intercellular space and tissue vacuolation were observed, and the number of inflammatory cells increased in the hepatopancreas (Figure 1E). On day 7 after exposure, the intercellular space of the hepatopancreas was reduced, but tissue vacuolation was still present (Figure 1F). On day 14 after exposure, the intercellular space and tissue vacuolation persisted and blood sinus dilation was observed in the hepatopancreas (Figure 1G). On day 21, the hepatopancreatic tissue structure was similar to that of the control group, indicating that it had largely recovered (Figure 1H).

### 3.3. Effects on Antioxidant Enzyme and Acetylcholinesterase Activities

**Figure 2:**
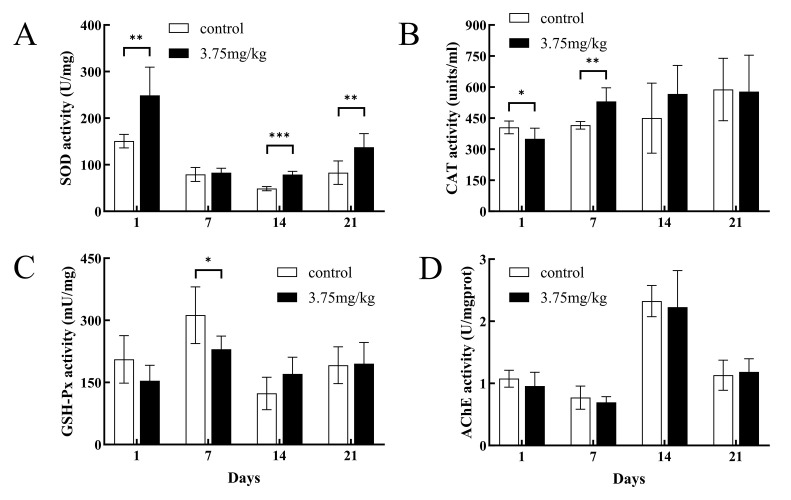
Effects of Cu^2+^ on SOD (**A**), CAT (**B**), GSH-Px (**C**), and AChE (**D**) enzyme activities in hepatopancreas. * *p* < 0.05, ** *p* < 0.01, *** *p* < 0.001 compared with the control.

In the Cu^2+^ exposure group, SOD activity in the hepatopancreas increased significantly on day 1 after exposure (*p* < 0.01), and returned to normal on day 7, but it increased significantly again on days 14 and 21 (*p* < 0.01) (Figure 2A). Compared to the control group, CAT activity in the hepatopancreas in the Cu^2+^ exposure group significantly decreased on day 1 (*p* < 0.05) but significantly increased on day 7 (*p* < 0.01) (Figure 2B). There was no significant difference in CAT activity between the treatment and control groups on days 14 and 21. GSH-Px activity in the hepatopancreas significantly decreased in the Cu^2+^-treated group compared to that in control group on day 7 (*p* < 0.05), but no significant differences were observed at the other time points (Figure 2C). Additionally, AChE activity in the hepatopancreas in the Cu^2+^-treated group did not significantly differ from that in the control group within the 21 days (Figure 2D).

### 3.4. Effects on Gene Expression

**Figure 3:**
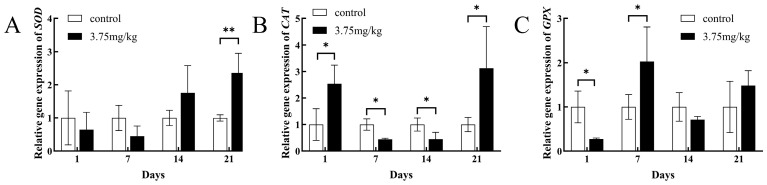
Effects of Cu^2+^ on the relative gene expression in tilapia hepatopancreas: *SOD* (**A**), *CAT* (**B**), and *GPX* (**C**). * *p* < 0.05, ** *p* < 0.01 compared with the control.

In the hepatopancreas in the Cu^2+^-treated group, the relative expression of the *SOD* gene was significantly up-regulated on day 21 (*p* < 0.01) (Figure 3A). Furthermore, the relative expression of the *CAT* gene was significantly up-regulated on day 1 (*p* < 0.05), significantly down-regulated on days 7 and 14 (*p* < 0.05), and significantly up-regulated again on day 21 (*p* < 0.05) (Figure 3B). Compared with the control group, the relative expression of the *GPX* gene in the hepatopancreas was significantly down-regulated on day 1 (*p* < 0.05) and significantly up-regulated on day 7 (*p* < 0.05), and then it returned to normal levels (Figure 3C).

## 4. Discussion

It is well known that, in fish, appropriate copper supplementation can improve their growth performance and feed utilization efficiency [27,28,29]. However, excessive intake of copper inhibits their growth and development [29,30,31]. In natural environments, mixing zones formed by the confluence of acid mine drainage (AMD) and neutral rivers exhibit significantly higher acute toxicity to fish compared to AMD alone, with copper being of the major toxic metals. Metals, as a result of their rapid precipitation in the mixing zones, coat fish gills, directly causing fish mortality, reducing fish survival and community diversity, blocking fish migration routes, and shrinking suitable habitats [32]. The Finniss River in northern Australia has long been impacted by mining pollution; elevated copper concentrations in natural waters have both acute lethal and chronic sublethal effects on fish, markedly reducing species diversity and abundance, altering the community structure, and eliminating sensitive taxa [33]. Wild *Hyphessobrycon luetkenii* inhabiting copper-contaminated streams in southern Brazil exhibit significant whole-body copper bioaccumulation, accompanied by systemic oxidative stress in the gills, brain, liver, and muscle due to elevated lipid peroxidation, protein carbonylation, and DNA damage. Although long-term exposed populations develop adaptive antioxidant homeostasis, they still sustain persistent physiological impairment and fitness costs [34]. In the present study, following copper exposure, the body mass, body length, and hepatopancreas mass of the tilapia increased gradually with developmental time, and no significant effects on growth performance were observed. This phenomenon can be largely explained by the temporary exposure to the copper through an intraperitoneal injection. At relatively safe concentrations, tilapia exhibits adequate detoxification capabilities. However, a similar study showed that exposure to copper at concentrations of 1–4 mg/kg via an intraperitoneal injection induced DNA damage in the sperm of adult *O. niloticus* males and caused severe morphological abnormalities in the hatched larvae of the next generation, including development arrest, cardiac edema, cyclopia, spinal abnormalities, craniofacial deformities, three eyes, and a reduced body length [25]. This suggests that tissues with a greater regenerative potential are able to gradually recover over time, whereas sensitive tissues such as those of the reproductive system sustain lasting damage. Therefore, morphological alterations alone are insufficient to fully characterize the organism’s response to environmental pollutants; thus, assessments using histological and molecular biological techniques are essential.

To characterize toxicological impacts directly at the cellular level, histological analysis is commonly performed on tissues including the liver, gill, intestine and muscle. The liver is the primary target organ of copper toxicity as it plays a dominant role in metabolism and detoxification [35]. Furthermore, the liver is also the main organ for copper accumulation in fish [36]. Accumulating evidence has revealed that exposure to copper induces dose- and time-dependent histopathological damage in hepatic tissue, predominantly characterized by hepatocellular vacuolization, fatty degeneration/hepatic lipidosis, sinusoidal dilatations, hyalinization, nuclear pyknosis, focal necrosis, and macrophage aggregate formation [37,38,39,40,41,42]. In this study, hepatopancreatic damage caused by copper included increased the intercellular space and tissue vacuolation, inflammatory cell infiltration, and blood sinus dilation, which is consistent with findings in *Seriola lalandi* [37], *Coregonus ussuriensis* [43], *Epinephelus coioides* [44] and *O. niloticus* [38]. Among these, sinusoidal dilatation is one of the most common liver lesions in fish following copper exposure. The increased intercellular space and tissue vacuolation indicated that the hepatopancreas tissue had lost its typical cellular structure. These histopathological changes may be associated with protein synthesis inhibition, energy loss, and substrate consumption, indicating hepatopancreatic dysfunction [43]. In addition, the increased infiltration of inflammatory cells into the hepatopancreas indicated that copper had induced inflammatory responses. However, these histopathological lesions were almost completely restored within 21 days post-exposure, suggesting a certain capacity for self-repair. In a study conducted by Jin et al. [37], the liver structure of *S. lalandi* had not fully recovered after a 14-day recovery period following 7 days of copper exposure. This may be attributed to the time-dependent nature of hepatic recovery as long-term exposure can compromise the liver’s ability to repair itself.

The toxicological mechanism of copper mainly involves cytotoxicity and immunosuppression [45]. This damage essentially results from toxic cascade reactions triggered by copper accumulation in which copper-induced oxidative stress serves as the driving force and plays a crucial role in activating inflammatory responses [27,46,47]. Excess copper in aquatic environments can cause injury in fish, leading to massive production of ROS and oxidative stress, ultimately disrupting normal cellular functions and causing organismal damage [48]. In addition, high ROS levels further damage mitochondria, leading to higher ROS production, creating a vicious cycle that sustains and amplifies oxidative stress [49]. To resist ROS-induced damage, organisms possess a series of antioxidant defense systems. SOD is a metalloprotein that can eliminate free radicals and serves as an important antioxidant by converting ROS into H_2_O_2_ and O_2_. CAT is a terminal oxidase that catalyzes the redox conversion of H_2_O_2_ into H_2_O and O_2_, thereby completing the antioxidant process. Glutathione is a naturally active peptide that exists in reduced (GSH) and oxidized (GSSG) forms. It scavenges free radicals and plays roles in liver protection, anticancer activity, and detoxification. GSH-Px can also catalyze the reduction of H_2_O_2_ to H_2_O and O_2_ using glutathione as an electron donor [50]. These enzymes exhibit tightly coordinated activity in response to oxidative stress.

In this study, SOD activity and the relative expression of the *SOD* gene in the hepatopancreas showed an increasing trend after copper exposure. A strong positive correlation was observed between enzyme activity and *SOD* gene expression, and their changes sensitively reflected intracellular redox status. On day 1, SOD activity was significantly elevated, suggesting that the hepatopancreatic antioxidant system was rapidly activated to scavenge the intracellular ROS produced in response to copper exposure [51]. Although SOD activity was not elevated on day 7 post-exposure, it increased on days 14 and 21, accompanied by upregulated relative expression of the *SOD* gene on day 21, indicating that the hepatopancreatic antioxidant system was still functional. This increasing trend is in agreement with the trends observed in previous investigations, which showed that copper sulfate induced significant increases in SOD activity in *Schizopygopsis younghusbandi* larvae [52] and that copper powder increased SOD activity in *Pangasianodon hypophthalmus* [39]. On the contrary, copper decreased SOD activity in juvenile *Macrobrachium nipponense* [53], *Pelteobagrus fulvidraco* [54], and *Carassius auratus* [55]. Therefore, the varied responses of SOD to copper are associated with the exposure route, duration and concentration, as well as species tolerance, all of which reflect the disruption of oxidative homeostasis in the organism.

In the present study, CAT activity only significantly changed on days 1 and 7 post-exposure. The decreased CAT activity on day 1 may be due to ROS-induced enzyme oxidation or substrate overload [56]. On day 7, with the recovery of hepatopancreatic function, the oxidative damage and substrate overload subsided. CAT activity increased to scavenge excess H_2_O_2_ and returned to baseline levels on days 14 and 21. In addition, the relative expression of the *CAT* gene was significantly up-regulated on days 1 and 21 but significantly down-regulated on days 7 and 14. Enzyme activity and gene expression often show asynchronous patterns in metal exposure studies; *CAT* gene expression typically rapidly increases during early exposure to cope with oxidative stress [57,58]. These results suggest possible post-transcriptional regulation and redox feedback mechanisms.

GSH-Px activity and *GPX* gene expression were temporally asynchronous on day 7, possibly due to the concurrent increase in CAT activity, as both enzymes function complementarily in removing H_2_O_2_ [59,60]. The majority of the changes in these enzyme activities and relative gene expression level were concentrated on days 1 and 7 after exposure, further confirming that the antioxidant system in the hepatopancreas was initially activated by the copper exposure. Meanwhile, significant changes in these three antioxidant enzymes are often accompanied by histological damage, such as cellular vacuolar degeneration, connective tissue disruption, fibrous tissue proliferation, and inflammatory cell aggregation in hepatopancreas [61,62,63]. Over time, both the enzyme activity and gene expression of the antioxidant defense system returned to baseline levels with the recovery of the histological structure of the hepatopancreas. As comprehensively reviewed by Lushchak (2014), changes in antioxidant enzyme activities and gene expression only reflect the activation of the defense system, not the actual magnitude of the oxidative damage to cellular macromolecules [64]. Antioxidant responses can be modulated by multiple factors independent of oxidative stress levels, and their activation does not necessarily correlate with the severity of oxidative injury. Our findings indicate a potential alleviation of oxidative stress by copper in tilapia.

Acetylcholinesterase (AChE) plays an important role in nerve impulse transmission by hydrolyzing acetylcholine into acetic acid and choline, but it must be rapidly degraded after release; otherwise, its accumulation blocks neural signaling [65]. Therefore, decreased AChE activity can impair nerve impulse conduction. Exposure to heavy metals can induce conformational changes in AChE, leading to altered activity; thus, AChE activity is considered an indicator of heavy metal toxicity [66]. In the present study, copper exposure did not cause significant changes in AChE activity in the hepatopancreas, suggesting that the toxicological effect was not primarily mediated by direct inhibition of AChE. Instead, the activation of the antioxidant defense system suggests that redox homeostasis was affected by the copper exposure. When redox homeostasis is impaired, oxidative stress ensues [64]. Therefore, although a direct measurement of ROS was not performed, considering the findings of previous studies [46,52,57,59], we speculate that oxidative stress is one of the important pathways in the mechanism of copper-induced toxicity in tilapia.

To achieve precise control over the exposure dose, intraperitoneal injection was employed for copper administration in this study. While this approach ensures exceptional dose accuracy and experimental reproducibility for mechanistic toxicological investigations, it cannot fully replicate the natural waterborne exposure routes encountered by fish in aquatic ecosystems, which may limit the direct extrapolation of our findings to field conditions. Our results clearly demonstrate that copper exposure activates the hepatic antioxidant system. However, key biomarkers of oxidative damage, such as malondialdehyde (MDA), protein carbonyls, and 8-hydroxy-2′-deoxyguanosine (8-OHdG), were not assessed. As emphasized by Lushchak (2014), this approach is insufficient to accurately determine the extent or temporal progression of oxidative stress as it cannot distinguish between activation of the antioxidant defense system and actual oxidative injury to cellular components [64]. In addition, we did not explore other potential toxicological pathways that may contribute to copper-induced hepatotoxicity. As such, the current dataset alone is insufficient to definitively establish oxidative stress as the primary mechanism underlying copper toxicity in tilapia.

## 5. Conclusions

In summary, short-term copper exposure via intraperitoneal injection induced significant time-dependent activation of the hepatopancreatic antioxidant defense system and histopathological damage in tilapia. These findings, combined with the absence of significant AChE activity changes, support a significant contribution of redox homeostasis to copper-induced hepatotoxicity. Following a 21-day recovery period, the antioxidant system largely returned to baseline, hepatopancreatic lesions were effectively repaired, and no sustained significant adverse effects on growth and development were detected. Further studies investigating additional toxicological pathways are required to fully elucidate the complete mechanism of copper toxicity in fish.

## Figures and Tables

**Table 1:** The effects of Cu^2+^ exposure on tilapia body length, body mass, hepatopancreas mass and hepatosomatic index.

Day	Body Length (cm)			Body Mass (g)			Hepatopancreas Mass (mg)			Hepatosomatic Index		
	Control	Cu^2+^	*p* -Value	Control	Cu^2+^	*p* -Value	Control	Cu^2+^	*p* -Value	Control	Cu^2+^	*p*-Value
1	3.28 ± 0.10	3.45 ± 0.08	0.245	3.39 ± 0.28	3.46 ± 0.25	0.850	71.43 ± 7.46	65.53 ± 7.83	0.598	0.024 ± 0.003	0.020 ± 0.03	0.396
7	3.47 ± 0.11	3.51 ± 0.09	0.791	4.24 ± 0.39	4.19 ± 0.31	0.921	63.09 ± 12.25	63.27 ± 6.80	0.989	0.019 ± 0.004	0.020 ± 0.003	0.804
14	3.72 ± 0.08	3.78 ± 0.17	0.740	4.80 ± 0.34	5.07 ± 0.46	0.590	108.11 ± 10.05	83.60 ± 10.01	0.099	0.025 ± 0.003	0.022 ± 0.005	0.581
21	3.87 ± 0.10	3.89 ± 0.69	0.927	5.98 ± 0.38	5.44 ± 0.30	0.272	98.32 ± 12.25	81.77 ± 5.51	0.223	0.014 ± 0.002	0.010 ± 0.002	0.224

## Data Availability

The original contributions presented in this study are included in the article/Supplementary Materials. Further inquiries can be directed to the corresponding authors.

## Supplementary Materials

The following supporting information can be downloaded at: https://www.mdpi.com/article/10.3390/toxics14050412/s1, Table S1: cDNA synthesis components; Table S2: RT-qPCR primer sequences. Figure S1: Environmental temperature changes during the experiment.
