# Supplementary material for: Physiological Responses and Post-Exposure Recovery of the Hepatopancreas in Nile Tilapia Following Copper Exposure
Source: Toxics. 2026 May 9;14(5):412. doi: 10.3390/toxics14050412 (PMC13211116; doi:10.3390/toxics14050412)
Supplement: Supplementary file 1 [file toxics-14-00412-s001.zip › toxics-4270218-supplementary.pdf]

Supplementary materials

1. Enzyme activity calculation formulas

$$C_{pr}(\text{g/L})=\frac{A_{sample}-A_{blank}}{A_{standard}-A_{blank}}\times C_{standard}\times N$$

$C_{standard}$: concentration of the standard solution, 0.524 g/L;
N: dilution factor of the sample prior to testing.

$$SOD\ inhibition\ rate(\%)=\left(1-\frac{A_{sample}-A_{sample\ blank}}{A_{control}-A_{control\ blank}}\right)\times 100\%$$

$$SOD\ activity(\text{U/mg}\ protein)=SOD\ inhibition\ rate(\%)\div 50\%\times\frac{V_{total}}{V_{sample}}\times N\div C_{pr}$$

V_{total}: total volume of the reaction system, 0.24 mL;
V_{sample}: volume of the sample added in the assay procedure, 0.02 mL;
N: dilution factor of the sample prior to testing (homogenization treatment is not counted as dilution);
C_{pr}: protein concentration of the sample homogenate, mg/mL.

$$CAT\ activity(\text{U/mg}\ protein)=\Delta A\times 271\div V_{sample}\div T\times N\div C_{pr}$$

271: calculation coefficient;
V_{sample}: volume of sample taken in this system, 0.1 mL;
T: reaction time, 60 seconds;
N: dilution factor of the sample prior to testing;
C_{pr}: protein concentration of the sample homogenate, mg/mL.

$$GSH-Px\ activity(\text{U/mg}\ protein)=\frac{A_{non-enzymatic}-A_{enzymatic}}{A_{standard}-A_{blank}}\times C_{standard}\times N\div T\div\left(V_{sample}\times C_{pr}\right)$$

$C_{standard}$: concentration of GSH standard solution in the color reaction, 20 μmol/L;
N: dilution factor of the enzymatic reaction system, 5 (2.5 mL/0.5 mL, fixed value);
T: enzymatic reaction time, 5 min;
V_{sample}: volume of sample taken in the enzymatic reaction, 0.2 mL;
C_{pr}: protein concentration of the sample homogenate, mg/mL.

$$AChE\ activity(\text{U/mg}\ protein)=\frac{A_{sample}-A_{control}}{A_{standard}-A_{blank}}\times C_{standard}\div C_{pr}$$

$C_{standard}$: concentration of the standard, 1μmol/mL;
C_{pr}: protein concentration of the sample homogenate, mg/mL.

2. Histopathological procedures

Hepatopancreas tissues of tilapia were fixed in 4% paraformaldehyde solution for 24h, and then dehydrated and hardened in a graded ethanol series as follows: 70%, 80 %, 90%, 95%, and 100% ethanol for 1h at each concentration. The tissues were then cl eared with a dewaxing agent (TO clearing agent) twice for 20min each to completely r eplace ethanol. Subsequently, the tissues were infiltrated in melted paraffin at 60℃ for 4h in an embedding machine to ensure sufficient penetration. The tissues were immedi

ately placed at the center of a preheated embedding mold using prewarmed forceps, a nd cooled to form solid paraffin blocks, which were labeled and stored at room tempe rature in the dark or at 4°C. The paraffin blocks were trimmed into a trapezoidal shap e and cut into 5-μm serial sections with a microtome. The sections were immediately mounted onto gelatin-coated glass slides and dried in an oven at 60°C for 4h until fir mly attached, followed by dewaxing and rehydration. The tissue sections were stained with hematoxylin and eosin (H&E) staining, sealed with neutral balsam, and dried at 37°C for 12h for long-term storage. Finally, images were captured and analyzed using an upright microscope.

3. Kit and conditions for cDNA synthesis

1. Add the following components to a DNase & RNase-free centrifuge tube:

Table S1. The kit for cDNA synthesis.

Reagent	Volume
RNA template	≤1 ug total RNA
2×StarScript III Buffer (with Primer)	10 μl
StarScript III RT Enzyme Mix	1 μl
Nuclease-free Water (DEPC-treated)	Bring to a final volume of 20 μl

2. Gently mix the contents and briefly centrifuge. Incubate at 50°C for 15 min.

3. Heat at 85°C for 5 min to inactivate StarScript III Enzyme Mix.

4. After the reaction, the obtained cDNA should be placed on ice for subsequent exper iments or stored frozen.

4. qPCR reaction program and primer sequences

The 20 μL PCR reaction system was established using 2×Taq PCR StarMix. The co mponents were as follows: 0.5 μL forward primer, 0.5 μL reverse primer, 1 μL cDNA template, 10 μL 2×RealStar Fast SYBR qPCR Mix, and 8 μL sterile water.

The three-step PCR amplification program was set as follows: initial denaturation at 95°C for 2min; followed by 40 cycles of denaturation at 95°C for 15s, annealing at 5 5°C for 30s, and extension at 72°C for 30s. After amplification, the PCR products were stored at 4°C. The sequences of the primers used in this study are provided in Table S 2.

Table S2. RT-qPCR primer sequences.

Gene	Forward primer	Reverse primer
β-actin	CAATGAGAGGTTCCGTTGC	AGGATTCCATACCAAGGAAGG
SOD	GGTGCCCTGGAGCCTA	ATGCGAAGTCTCACTGTC
GPX	CCAAGAGAACTGCAAGACGA	CAGGACACGTCATCCTACAC
CAT	TCCTGAATGAGGAGGCGA	ATCTTAGATGGGTGATG

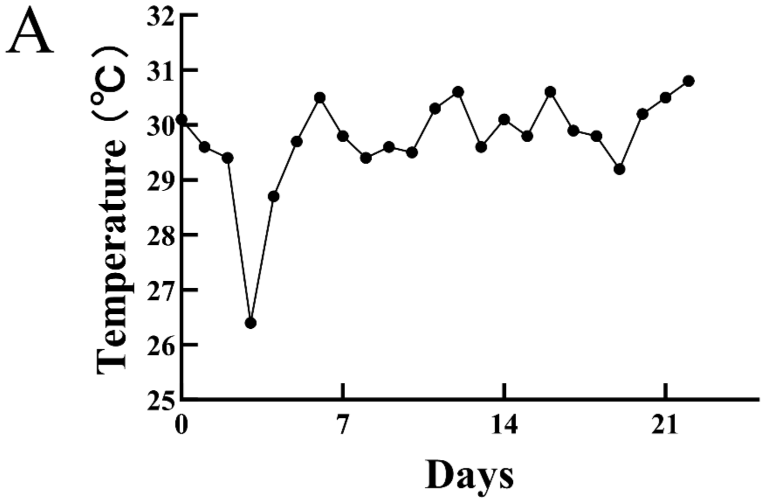

Figure S1: Environmental temperature changes during the experiment.
